# COVID-19 distress affects healthcare and administrative workers equally at a tertiary hospital center in Brazil

**DOI:** 10.47626/1679-4435-2022-862

**Published:** 2022-03-30

**Authors:** Ana Rita Dias Resende, Bianca Cavalca Dedini, Flávia Da Silva Domingos Santos, Giuliana Gisele Magalhães, Giovana Fiod Grela, Samuel Servinhani Fernandes, Ana Carolina Gonçalves Olmos, Marilia Capuço Oliveira, Gerardo Maria Araújo-Filho

**Affiliations:** 1 Departamento de Psiquiatria e Psicologia Médica, Faculdade de Medicina de São José do Rio Preto, São José do Rio Preto, SP, Brazil.

**Keywords:** COVID-19, epidemiology, pandemics, psychiatry, health personnel

## Abstract

**Introduction:**

The COVID-19 outbreak exposes healthcare workers to an increased risk of distress and psychiatric symptoms.

**Objectives:**

To evaluate psychological suffering and mental disorders among healthcare workers at a tertiary hospital, a referral center for COVID-19 treatment.

**Methods:**

An observational, cross-sectional, quantitative study with descriptive methodology. Fifty-eight healthcare workers who attended consultations at the hospital’s Mental Health Outpatient Clinic were included. The study was carried out after approval by the research ethics committee at the Faculdade de Medicina de São José do Rio Preto (32665020.3.0000.5415).

**Results:**

81% were women, mean age was 38.98±10.6 years, 20 (34.5%) were administrative staff, 24 (41.4%) were attending a first consultation, and 28 had had previous psychiatric attention at other services. Sixteen (28%) reported new symptoms during the pandemic, with anxious (10), irritable (3), and depressive (2) symptoms being the most frequent. Anxiety (26) and depressive disorders (19) were the most prevalent. As for exposure to news, the most common feelings were fear (19) and anguish or concern (9). The most common feelings associated with the pandemic were fear and recurrent thoughts of social and economic impact (27). The main reflections were about the meaning of life (17), human vulnerability (11), and the importance of the family (7). Regarding prospects for the future, 70.7% (41) reported hope for improvement.

**Conclusions:**

Initial data suggest a high prevalence of anxiety and depressive symptoms, as well as sleep disturbances, regardless of work team. Fear of death and uncertainty about the future are also prevalent. These data reinforce the importance of developing strategies to reduce the risks to this population’s mental health.

## Introduction

Crises and life-threatening situations usually affect everyone in different ways, but one element common to all who go through them is uncertainty regarding the future. COVID-19 identified in China at the end of December 2019, quickly spread across the world and due to the high rate of contagion and the trail of infected and dead people, has us facing an imminent life-threatening situation.^1,2^

Often during such situations, it is common for the medical and scientific community to focus on practical issues such as pathophysiological mechanisms and preventive and treatment measures,^1,2^ but the important negative impact on mental health cannot be ignored. For healthcare workers, especially doctors and nurses who are on the front line, the impact can be worrying. The increased workload, the responsibility, and the fear of being infected and contaminating family members can trigger emergence of psychiatric symptoms or aggravate pre-existing disorders.^1,3,4^ In addition to work-related problems, these workers need to deal with the psychological effects of social isolation, loss of their support networks, and the stigma of being in direct contact with the risk of infection. They may experience disturbed sleep, anxiety, feelings of hopelessness, or worsening of depressive and anxious conditions, with an increased suicide risk. The risk of increasing consumption of legal or illegal psychoactive substances should also be considered.^2,4-6^

Several studies have demonstrate that healthcare workers have an incidence of psychiatric disorders up to three times higher than the general population, which contrasts, however, with a low rate of seeking help.^7,8^ In the last few months, several studies have reported the presence of psychological stressors in mental health workers, with a higher incidence of depressive and anxious symptoms and sleep disorders when compared to the general population.^9-12^ Also, higher levels of fear, anxiety, and depressive symptoms are observed in the medical team when compared to the administrative team.^13^ Based on these premises, the present study aimed to assess the presence of psychological distress and mental disorders among healthcare workers during the COVID-19 pandemic, in order to investigate the presence of new symptoms and their relationship to the pandemic, as well as feelings and fears evoked during this period.

## Methods

This is an observational, cross-sectional, quantitative study with descriptive methodology. The present study was carried out after approval by the research ethics committee at the Faculdade de Medicina de São José do Rio Preto (FAMERP) under protocol 32665020.3.0000.5415. All participants signed a free and informed consent form.

### Sample selection

The FAMERP Hospital Center is one of the largest teaching hospitals in Brazil. It receives direct referrals from a region with more than 2 million inhabitants and plays a central role in the care of patients with suspected or confirmed COVID-19. Participants were sampled from a Mental Health Outpatient Clinic located at the center that is specialized in providing mental health treatment to workers from the hospital. We offer psychological and psychiatric care within a free demand model with an average of 12 patients per week, 600 per year, which can vary according to the demand. Data collection was carried out through clinical interviews with patients scheduled from May to August 2020 at the Outpatient Clinic.

### Procedures

Sociodemographic characteristics analyzed included age, sex (male/female), marital status, religion, educational level, occupation (healthcare staff versus administrative staff), close contact with infected patients, and history of quarantine from family. Clinical characteristics included lifetime psychiatric treatment, positive diagnosis of COVID-19, presence of new psychiatric symptoms since the start of the severe acute respiratory syndrome coronavirus 2 (SARS-CoV-2) outbreak, recurrence or relapse of previous psychiatric disorders, lifetime drugs and alcohol use/history of increased use in pandemic period, social media exposure, social support, leisure time, and feelings, fears, and reflections about life and the pandemic.

### Statistical analysis

All statistical analyses were performed using the SPSS software package (version 20). The data were grouped using a systematic data collection form, and values of p < 0.05 were considered significant.

## Results

Fifty-eight workers from the Hospital Center who attended the FAMERP Mental Health Outpatient Clinic during the period participated in the study. Of these participants, 47 (81%) were women and the average age was 38.98±10.66 years, ranging from 19 to 67 years. Regarding other sociodemographic characteristics, the majority (31; 53.4%) reported between 10 to 13 years of study, 50% (29) were married, 68.3% (39) had at least one child, and the most prevalent religion was Christianity (46; 79.3%).

The occupations of the employees were classified as healthcare staff (38; 65.5%) or administrative staff (20; 34.5%). Among the healthcare staff, 68.5% were from the nursing team, namely: nursing technician (15; 57.7%), nursing assistant (8; 30.7%), and nurses (2; 11.6%). Most were working at the time of the assessment (49; 84.5%), and nine (15.5%) reported working directly with patients infected with COVID-19. Four (6.9%) participants reported having a positive COVID-19 diagnosis since the beginning of the pandemic.

Most of the participants (36; 62%) referred no close contact with relatives or friends at high risk from COVID-19 infection. The preexisting risk conditions reported by those who did report contact were advanced age (12; 54.5%) followed by cardiac conditions (6; 27.3%), pneumopathies (5; 22.7%), diabetes (5; 22.7%), and obesity (1; 4.5%). Twenty individuals quarantined from their families for long periods of time.

Out of the professionals who participated in the survey, 28 (48.3%) reported having received previous psychiatric attention at other services. Out of all the interviews conducted, 24 (41.4%) were attending their first consultation. Sixteen individuals (28%) reported having new symptoms since the beginning of the pandemic, with anxiety (10; 62.5%) being the most frequent, followed by irritability (3; 18.8%), depressive symptoms (2; 12.5%), sleeping disorders (1; 6.2%), and eating disorders (1; 6.2%). Nine patients (32.15%) had relapse of symptoms in the period, and 15 (53.4%) had recurrence of a psychiatric condition. Twenty-seven (46.5%) patients reported a correlation between current symptoms and the period of the pandemic, due to fear of contagion and/or isolation. The new symptoms were statistically more prevalent among those who were attending their first consultation, those who had been stable and had a recurrence, and those who considered that the current symptoms were due to the pandemic, as shown in Table 1. There were no statistically significant correlations between appearance of new symptoms and work team (administrative or healthcare).

**Table 1:** Occurrence of new symptoms and significantly correlated variables

	New symptoms (n)		p-value
	No	Yes	
First consultation			
No	28	6	0.044*
Yes	14	10	
Recurrence in previously stable patient			
No	41	8	≤ 0.001*^†^
Yes	1	8	
Current symptoms due to pandemic			
No	27	4	0.007*
Yes	15	12	

* Statistically significant association, adopting a significance level of 5%.

† Fisher’s test, the other analyses were performed using the chi-square test.

**Figure 1:**
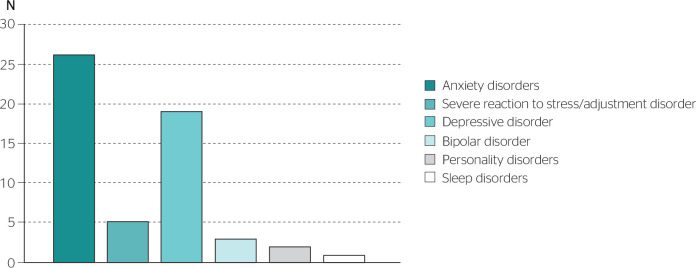
Psychiatric diagnosis at current consultation.

Anxiety disorders (26; 44.9%) and depressive disorders (19; 32.8%) were the most prevalent psychiatric diagnoses made during the consultations (Figure 1). It is worth emphasizing that all patients evaluated had a psychiatric diagnosis.

Tobacco use was reported by six participants (10.4%), use of alcoholic beverages by 12 (20.7%), and dependence on another psychoactive substance was reported by one (1.7%), while only two (10.5%) individuals reported having increased consumption. Antidepressants (38), antipsychotics (8), and benzodiazepines (7) were the most prevalent psychotropic prescription drugs in use.

Regarding media exposure, 53 patients (91.4%) reported being able to restrict it (do not watch or select media outlets and topics). When exposed to news on the topic, the most common feelings were fear (19; 32.8%), followed by indifference or anguish or concern (9; 15.5%), skepticism (7; 12%), and sadness (6; 10.4%). Considering the amount of time spent daily on the subject of COVID-19, the majority referred to it as “average” (20; 34.5%) followed by little or very little. Only 13.8% considered that this theme was occupying their lives for “too much time” on a daily basis.

The most common feelings related to the pandemic were fear and recurrent thoughts about the social and economic impact (27; 46.5%), followed by loss of faith in society and search for religious explanations (8; 13.8%), minimizing the situation or believing it to be a political maneuver (8; 13.8%), hope (6; 10.4%), and uncertainty (3; 5.2%). Regarding fears, most participants reported fear related to their own death or the death of loved ones (24; 41.4%) and fear of being contaminated or contaminating others (21; 36.2%), in addition to fear of the consequences of the disease (9; 15.5%).

The main reflections were about the meaning of life (gratitude, opportunity for growth, unity among people) (17; 29.3%), human vulnerability (11; 19%), the importance of family (7; 12%), the need for humanity to evolve (6; 10.4%), religion (2; 3.5%), and fear of death (1; 1.7%). About 24% of the sample reported not having reflected on anything during the period or did not know how to answer.

Regarding the prospects for the future, 41 (70.7%) reported hope for improvement. Among the others, 14 (24%) individuals presented negative speech permeated by uncertainty and hopelessness and three (5.2%) of them said they did not know what to expect.

When asked about maintenance of leisure and relaxation activities, 27.5% (16) of the sample reported not doing any activity. Among the others, the main activities were related to films/series/music (21; 50%) followed by physical activity (9; 21.5%) and religion (8; 19%). 24.2% (14) of the individuals did not have a support network.

Table 2 shows the analysis of associations between the variable “first patient visit” and variables of interest that were significant.

**Table 2:** Associations between sociodemographic and clinical characteristics and status of consultation (first visit versus follow-up)

	First consultation (n)		p-value
	No	Yes	
Marital status			
Married/stable relationship	13	16	0.033*
Other	21	8	
Contact with risk group			
No	25	11	0.032*
Yes	9	13	
New symptoms			
No	28	14	0.044*
Yes	6	10	
Current symptoms due to pandemic			
No	24	7	0.002*
Yes	10	17	
Current symptoms due to fear of contagion			
No	30	10	≤ 0.001*
Yes	4	14	
Current symptoms due to impact of isolation			
No	28	14	0.044*
Yes	6	10	
Need to stay away from family			
No	27	11	0.008*
Yes	7	13	
How much the subject (COVID-19) took over the patient’s life			
Little/very little	14	3	0.003*
Average/medium	16	9	
A lot/completely	4	12	
Feelings related to the news			
None, politics, outrage	15	3	0.010*
Fear, sadness, worry, anguish	19	21	
Works directly with COVID-19			
No	32	17	0.026*^†^
Yes	2	7	
Diagnosis of COVID-19			
No	34	20	0.025*^†^
Yes	0	4	
Psychotropic prescription after current consultation			
No and/or no drugs alteration	25	6	≤ 0.001*
Yes and/or with drugs alteration	9	18	

* Statistically significant association, adopting a significance level of 5%.

† Fisher’s test, the other analyses were performed using the chi-square test.

Proportionally, the most prevalent categories among individuals who went to the first consultation were: married/in a stable relationship, had contact with people from the risk group, believed to have new symptoms resulting from the pandemic, had to be apart from their families, had feelings about the news tending to fear, sadness, worry and anguish, worked directly with COVID-19, had a diagnosis of COVID-19, and considered that this subject (COVID-19) took a lot of their time/completely took over their lives. It is worth clarifying that prescriptions of psychotropic drugs were more frequent among individuals who were consulting for the first time.

## Discussion

We found that healthcare workers were experiencing high rates of anxiety and depression, which was similar to other studies, with no significant differences, however, between healthcare and administrative staff. Moreover, our study showed that presence of new psychiatric symptoms had no correlation with being in one or another group. Thus, the mental health burden of the outbreak impacts everyone in the hospital center, no matter the job or workplace. These data differ from literature which suggest different prevalence rates in medical versus nonmedical workers,^14,15^ frontline workers,^16^ and job title.^2^ This difference may be partially explained by the fact that our participants were working at a tertiary hospital with a complex organized structure for mental healthcare. Our service offers regular psychiatry and psychology assistance for professionals with face-to-face or online consultations and the service is easily accessed by the workers themselves or their team leaders.

The findings suggesting an impact of the pandemic on mental health are similar to data in the literature. Previous experience in other epidemic periods yields evidence of significant damage to mental health. Tansey et al.^17^ reported that there were three times more consultations with psychiatrists than with infectologists in the 2003 SARS epidemic. Several authors report increased levels of depressive symptoms, anxiety, psychosis, and suicide in epidemic periods.^18,19^ Torales et al.^19^ cites evidence of increased psychiatric symptoms such as anxiety and post-traumatic stress in health workers in the SARS-CoV epidemic of 2003 in Singapore and Taiwan, Ebola in 2014 in Sierra Leone, SARS-CoV in Korea in 2015, and Ebola in Congo in 2018. Studies carried out at the beginning of the SARS-CoV-2 epidemic in China described moderate to severe psychological impact in 53.8% of individuals.^2^ Meng et al.^20^ found high rates of anxiety and depressive symptoms among doctors in Chinese hospitals.

Our study showed that 48% (28) of the sample was already receiving psychiatric attention and there was recurrence of the previous condition in 53.4% of patients. This is similar to reports in the literature and it is noteworthy that regardless of the presence or absence of a pandemic, healthcare workers have three times more mental disorders than the general population^20^ and situations of intense overload such as the current context make these professionals even more susceptible,^21,22^ demanding strategies to reduce the psychological impact.

When considering the sociodemographic variables, the fact that most of the sample is female is similar to other studies. Some authors suggest women have greater vulnerability to mental disorders,^19,20,23^ but also highlight the possibility of bias due to the greater number of respondents being female, in addition to being more exposed because of their workplace.^23^ It is worth mentioning that our sample comprised employees who voluntarily sought the clinic and that, for cultural reasons, women tend to seek help earlier. Further studies should be designed to actively search for cases and evaluate this possible correlation.

The pandemic provokes countless feelings and reflections, the most recurrent of which are fear and uncertainty about the future, which is similar to our findings. These feelings negatively impact on mental health, increasing the risk of anxiety, depressive symptoms, somatization, and increased consumption of alcohol and tobacco.^5,24-27^ We should also highlight that frequent use of social media and the excess of information may contribute to further aggravating the psychological consequences of the pandemic.^19,28-30^ In our sample, it can be observed that even though most of the subjects were controlling their exposure, when exposed, negative feelings are mobilized and can contribute to emergence of psychological distress. It is therefore important to implement mental health actions focusing on addressing these feelings, such as psychotherapy, support groups, and listening techniques, besides psychoeducation on the harms of social media.

Our service registered a 36% increase in demand from February 2020 to February 2021, but considering the total number of employees and workers, demand is still low. Strategies to publicize the mental health support service, psychoeducation campaigns focusing on risk groups, and active screening of individuals in mental distress are necessary to mitigate the psychological effects of this pandemic.

Limitations of this study include the fact that our institution is a tertiary center with a good structure for mental health which differs from most centers in Brazil. Also, these data were obtained from patients who voluntarily sought the outpatient clinic, which may be a source of bias. Moreover, the study was performed early in the outbreak and a follow-up study should be conducted to assess long-lasting effects, especially now with the increasing number of infections and deaths.

## Conclusion

Healthcare workers are exposed to increased risk of psychological distress and mental illness during the pandemic, regardless of whether they are members of the administrative or healthcare teams. Initial data suggest a high prevalence of anxiety and depressive symptoms, as well as sleep disturbances. Fear of death and uncertainty about the future are also prevalent. These data reinforce the importance of developing strategies to reduce the risks to this population’s mental health. Follow-up studies are necessary to better understand the effects of the pandemic on health care workers and large-scale studies should be conducted to assess the long-term mental health impact of COVID-19.
